# Inequitable gender norms and its associated factors among university students in southern Ethiopia: a cross-sectional study, 2022

**DOI:** 10.3389/fpubh.2024.1462782

**Published:** 2024-12-19

**Authors:** Yirgalem Tola Kelecha, Amanuel Albene Ayele, Habtamu Samuel Goda, Mesarch Hailu Demissie, Temesgen Mohammed Toma

**Affiliations:** ^1^Department of Midwifery, Arba Minch College of Health Sciences, Arba Minch, Ethiopia; ^2^Department of Malaria and Neglected Tropical Diseases, Armauer Hansen Research Institute, Addis Ababa, Ethiopia; ^3^Department of Public Health, Arba Minch College of Health Sciences, Arba Minch, Ethiopia; ^4^Department of Health Information Technology, Arba Minch College of Health Sciences, Arba Minch, Ethiopia; ^5^CDC Project Regional HIV Case Surveillance Coordinator, Public Health Emergency Management Directorate, South Ethiopia Region Health Bureau Public Health Institute, Jinka, Ethiopia

**Keywords:** gender equality, gender norm, inequitables gender norm, inequitable, university students

## Abstract

**Background:**

Gender defined as the socially constructed roles, behaviors, activities, and characteristics that society deems appropriate for men, women, and other gender identities. Inequitable gender norms promote male dominance and aggressiveness while portraying women as being subservient. Ensuring equitable gender norms is a prerequisite for achieving gender equality in a society. The rise in maternal mortality and morbidity, adolescent pregnancies, unwanted pregnancies, unsafe abortions, HIV/AIDS, gender-based violence, and obstacles to reproductive health care are all linked to inequitable gender norms. On the other hand, not much is known regarding inequitable gender norms and their correlation in our setting.

**Objective:**

The purpose of this study was to evaluate university students’ attitudes regarding inequitable gender norms and responsible factors.

**Design:**

Between October 25 and November 10, 2022, students from Jinka and Arba Minch University participated in an institution-based cross-sectional study.

**Methods:**

A total of 635 students were selected using a multi-stage sampling technique. The Gender Equitable Men Scale was employed to evaluate inequitable gender norms. Data were verified and entered into Epi-Data Version 3.1, then analyzed using SPSS Version 25.0. Binary logistic regression was initially used to identify potential factors associated with inequitable gender norms, variables having a *p*-value <0.25 in bivariate analysis considered for multivariable regression. The final model’s fit was assessed using the Hosmer–Lemeshow goodness-of-fit statistic, confirming its adequacy. Statistical significance was determined at a threshold of *p* < 0.05.

**Results:**

Of the study participants, 44.1% (95% CI: 40.10, 48.10%) had an inequitable gender norm, while the mean score for a inequitable gender norm was 61.4 (SD 8.4). Inequitable gender norm was associated with sex being male (AOR = 1.75, 95%CI: 1.19, 2.56), romantic relationship breakup (AOR = 2.10, 95% CI: 1.14, 3.99), and having a negative attitude toward gender equality (AOR = 3,14, 95% CI: 2.15, 4.58).

**Conclusion:**

A notable number of participants expressed support for inequitable gender norms. This underscores the importance of comprehensive efforts by relevant stakeholders to address identified factors and promote equitable gender norms among university students.

## Background

Gender is the socially constructed roles, behaviors, activities, and characteristics that society considers appropriate for men, women, and other gender identities, in contrast to sex, which refers to biological traits. Gender must includes a spectrum of identities, such as non-binary, transgender, and gender-fluid, reflecting the diverse ways individuals experience and express gender within their cultural and social contexts (1). However in Ethiopia, cultural, religious, and legal conventions firmly establish the binary understanding of gender as male and female, making the inclusion of other genders a delicate topic.

Social expectations that specify how people of different genders should act, seem, and feel what is seen suitable for men, women, boys, and girls are known as gender norms (2). Gender norms, whether equitable or inequitable, shape power dynamics and responsibilities (3, 4). Inequitable gender norms favor men over women, creating unequal power dynamics and disparities in opportunities, resources, and rights. Rooted in institutions like family, education, media, and religion, they harm economic, educational, and health outcomes while marginalizing diverse gender identities (5). In sub-Saharan Africa, inequitable gender norms exacerbate existing barriers to women’s healthcare access, education, limited income, inadequate transportation, and poorly resourced health systems. These disparities lead to a disproportionate burden of disease on women, particularly in obstetric and neonatal health (6). This imbalance can significantly impact women’s overall quality of life, including their physical, mental, and reproductive health (7, 8).

Gender socialization occurs throughout time and space, starting from birth, where individuals are shaped to conform to established norms either directly or indirectly (9). The gender norm individuals adopt through gender socialization, regarding both equity and identity, are influenced by various factors at different levels (macro, meso and micro level) (10). These include broader societal influences (such as socio-economic conditions, political and social structures), intermediate influences (like family, peers, social networks, and institutions), and individual factors (such as biological sex, cognitive development, and physical and sexual maturation) (11).

In societies lacking gender equality like Ethiopia, prevalent gender norms often result in harmful public health consequences, including increased maternal mortality and morbidity, adolescent pregnancy, unintended pregnancies, unsafe abortions, higher rates of HIV/AIDS, perpetuation of gender-based violence, and barriers to accessing sexual and reproductive health services (12, 13).

Women, girls, and individuals of diverse gender identities often encounter heightened gendered risks, restricting their access to social, educational, economic, and political opportunities, as well as essential health services (14, 15). Rigid gender norms contribute to harmful behaviors toward women, with public health research showing that men who strongly embrace traditional notions of masculinity and dominance are more likely to engage in sexual assault and domestic violence, exacerbating health inequities and increasing the burden on healthcare systems (16, 17). For example in Ethiopia, there is widespread acceptance of inequitable gender norms. According to the 2016 Ethiopian Demographic and Health Survey, 63% of women and 28% of men believe that beating a wife for any reason is justified. Additionally, the prevalence of spousal physical, sexual, or emotional violence in Ethiopia stands at 38% (18).

In Ethiopia, the higher education sector faces a substantial gender gap. For example, in the 2017/18 academic year, the proportion of female students was 34.53% at the undergraduate level, 16.6% at the second-degree level, and 9.7% at the third-degree level (19, 20). Students from low socioeconomic backgrounds in Ethiopia face financial barriers to higher education, struggling to afford tuition, housing, and other costs. These challenges often prevent them from enrolling or continuing their studies (21).

Therefore, this study aims to explore inequitable gender norms among university students in Ethiopia, an area with limited research. By examining the factors that influence these norms, the study seeks to understand students’ attitudes, behaviors, and interactions. The findings will provide valuable insights to guide interventions, policies, and educational strategies that promote gender equity, helping to create a more inclusive and equitable environment within universities. This research is important for shaping the future leaders of Ethiopia, who will play a crucial role in addressing gender inequality in the broader society.

## Methods

### Study design, period, and setting

A school-based cross-sectional study was conducted among students of Arba Minch and Jinka Universities from October 25 to November 10, 2022. Arba Minch University, located 505 kilometers southwest of Addis Ababa, has a student body of 12,633 undergraduates across several academic units including Technology, Natural Science, Business and Economics, Medicine and Health Science, Social Science and Humanities, and Agricultural Science. Jinka University, situated 737 kilometers south of Addis Ababa in Jinka town, hosts 4,385 students in programs spanning Social Sciences and Humanities, Business and Economics, Agricultural Sciences, Computational and Natural Sciences, Health Sciences, and Law. Students from diverse backgrounds across Ethiopia attend these universities.

### Population

The source population for this study included all undergraduate students at Arba Minch and Jinka Universities. From this population, we selected students randomly from each department at both universities to form our study population.

### Inclusion and exclusion criteria

Students who were engaged in field practices, critically ill, or on semester break during the study period were excluded from participation.

### Sample size determination

The sample size was determined assuming a 50% proportion of inequitable gender norms, as no previous studies had addressed this issue within the same study groups. The calculation used the single population proportion formula with a 95% confidence interval and a 5% margin of error.


$$n\;=\;\frac{{\left(Z\left(1-\frac{\alpha }{2}\right)\right)}^{2}\times p\times \left(1-P\right)}{d^{2}}\;=\;\frac{\left(1.96^{2}\right)\times \left(0.50\times 0.50\right)}{0.05^{2}}=384.$$

After accounting for a design effect of 1.5 and a 10% non-response rate, 635 students were selected for the study. Where n is the required sample size, Z*α*/2 = critical value for normal distribution at 95% confidence level which equals 1.96 (*Z*-value at *α* = 0.05), *p* = proportion students with inequitable gender norm of 50%, d = margin of error of 5%.

### Sampling procedure

A multi-stage sampling approach was employed to select 635 students from Arba Minch and Jinka Universities. First, 22 departments (17 from Arba Minch University out of 108 and 5 out of 14 from Jinka University) were selected using a lottery method. From Arba Minch University, 9 natural science departments were included: Biology, Chemistry, Physics, nursing, Computer Science, Environmental Science, Civil Engineering, and Electrical Engineering. Additionally, 8 social science departments were selected: Sociology, Psychology, Management, Economics, Political Science, History, Geography, and English Literature.

From Jinka University, the 3 natural science departments included were Agriculture, Natural Resource Management, and Animal Science, while the 2 social science departments were Social Work and Business Management. The sample size was then proportionally allocated to each department based on student population. A sampling frame was created by organizing students’ registration numbers by academic year. Finally, simple random sampling was applied using computer-generated methods to randomly select students from each academic year within the selected departments. The surveys evaluated gender norms, demographic information, equality, and contextual factors using self-administered questions. Randomness was guaranteed by a multi-stage sampling procedure that included proportional sample distribution, computer-generated random student selection, and lottery-based department selection.

### Definitions and measurement

#### Inequitable gender norm

The 24-item Gender Equitable Men (GEM) scale was employed to categorize gender norms. First itemsnegatively worded (indicating gender-inequitable attitudes) were reverse-coded. Then participants’ responses were scored as follows: Agree = 1; Partially Agree = 2; Do Not Agree = 3. Each participant’s scores for the GEM scale items were summed to create a composite discrete variable. This composite score was then categorized into two groups: inequitable gender norm score (22–37) and equitable gender norm score (40–72) (38).

#### Physical violence during childhood

Six questions were asked about experiences of physical violence, defined as being slapped or having objects thrown at you that could cause harm, being pushed or shoved, being hit with a fist or another object that could hurt, being jerked, hauled, or beaten, and/or being threatened with a gun, knife, or other weapon. A student is considered to have experienced physical violence if they respond affirmatively to at least one of these questions from birth up to 18 years of age.

#### Household decision-making power

The composite score comprises six questions designed to assess the degree of women’s involvement in decision-making within the home during childhood. Each question offers three response options: another family member (including the father only), joint decision-making, and the woman (mother) deciding alone. Responses were scored as follows: another family member decides = 0, joint decision-making = 1, and the woman (mother) decides alone = 2. After calculating the total score, a score above the mean indicates good household decision-making power (39).

#### Gender equality attitude

Gender equality attitude was evaluated based on participants’ responses to six specific questions. Negatively stated questions were reverse-coded during data processing. The responses were then summed up, and a score above the mean indicated good knowledge about gender equality.

### Data collection tool and procedures

A structured self-administered questionnaire was used to collect data on socio-demographic characteristics, childhood experiences, behavioral factors, media usage, and gender equality attitudes. The questionnaire was developed by reviewing various literature sources (9, 11). The Gender Equitable Men scale, originally developed by Pulerwitz et al. (3), encompasses various psychometric domains related to gender norms, including gender-based violence, reproductive health and disease prevention, sexuality, domestic life, and childcare. The modified Gender Equitable Men scale is utilized in Ethiopia (22, 23). An example of a minor change includes altering an attitude statement about sexuality from “It is the man who decides what type of sex to have” to “It is the man who decides when to have sex with a partner.” The internal consistency of the scale has been assessed using Cronbach’s alpha, yielding a Cronbach’s alpha coefficient of 0.81 in Ethiopia (23).

Before consenting to data collection, each participant received comprehensive information on the study’s objectives, including the fact that participation was voluntary, that withdrawal was possible at any moment without repercussions, and that confidentiality would be guaranteed. The questionnaire was administered to participants after gathering them in a classroom setting to prevent information exchange among students. Supervisorswho were hired to oversee the data collection process closely monitored the entire data collection process on a daily basis.

### Data quality assurance

A structured questionnaire was first prepared in English and then translated into Amharic. To ensure accuracy and consistency in translation, it was subsequently back-translated into English by different translators. This process aimed to identify and rectify any discrepancies that may have arisen during translation. The Gender Equitable Men scale, consisting of 24 questions, has been validated in various sub-Saharan countries to assess attitudes towards gender norms. In Ethiopia, validation of this tool was conducted by Horizons research, ensuring its appropriateness and reliability for use in local contexts (24). Before beginning data collection, the internal consistency of the scale was assessed using Cronbach’s alpha on a subset comprising 10% of the sample (64 students) at Wolaita Sodo University, yielding a Cronbach’s alpha coefficient of 0.78. Throughout the data collection process, supervisors provided oversight to ensure accuracy and adherence to protocols. Following data collection, thorough reviews were conducted to check for completeness and consistency. This quality assurance step ensured that all required information was gathered accurately before proceeding with data entry.

### Data processing and analysis

Following data collection, the collected data were meticulously checked and entered into Epi-data software version 3.1. Subsequently, the data were exported to SPSS version 25 for comprehensive data cleaning and analysis. Descriptive statistics were computed for all variables based on their scaling. Continuous variables were summarized using mean and standard deviation, while categorical variables were assessed using frequencies and proportions. To examine differences in responses to gender norms items between male and female students, stratification by sex was conducted, and statistical differences were determined using the chi-square test. To identify factors associated with inequitable gender norms, a binary logistic regression model was employed. Crude Odds Ratios (COR) with 95% confidence intervals (CI) were used to present the results of the bivariable analysis. The multivariable model included all variables that had a significance level of *p* < 0.25 in the crude regression analyses to identify independent factors influencing gender inequitable norms. The strength of association was determined by Adjusted Odds Ratios (AOR) and reported with a 95% CI. A significance level of *p* < 0.05 was used to declare statistical significance. The Hosmer–Lemeshow goodness-of-fit statistic was satisfied with a *p*-value of 0.39, indicating good model fit. Multicollinearity among covariates was assessed using the variance inflation factor (VIF), with the highest observed VIF value being 2.37, indicating no significant multicollinearity issues.

## Results

### Socio-demographic and economic characteristics

A total of 615 students completed the questionnaire in the current study, yielding a response rate of 97%. The mean age of respondents was 22.5 (SD = ±2.5), and most of them, 491 (79.2%), belong to the age group 21–25. In this study, 239 (39.0%) participants were female. Of the study participants, 80 (13.0%) were married and 115 (18.7%) claim to be in a relationship currently, whereas 92 (15.0%), had a romantic relationship breakup (Table 1).

**Table 1:** Socio-demographic characteristics of study participants at Jinka and Arba Minch University, Southern Ethiopia, 2022.

Variables	Category	Frequency (*n*)	Percent (%)
Age (in year)	= < 20	81	13.2
	21–25	491	79.2
	>25	43	7.0
Sex	Male	376	61.0
	Female	239	39.0
Marital status	Married	80	13.0
	Never married	535	87.0
Relationship status	In relationship	115	18.7
	Was in relationship	92	15.0
	Never in relationship	328	53.3
Field of study	Social science	214	34.8
	Natural science	401	65.2
Year of Study	First-year	83	13.5
	Second year	198	32.2
	Third year	253	41.1
	Four and above	81	13.2
Place of residence before university	Urban	369	60.0
	Rural	246	40.0
School attended at preparatory	Private	117	19.0
	Public	498	81.0
Mother’s educational status*	No formal education	196	31.9
	Primary	227	36.9
	Secondary	82	13.3
	Above secondary	110	17.9
Father’s educational status**	No formal education	162	26.3
	Primary	159	25.9
	Secondary	105	17.1
	Above secondary	181	30.7

*Mother means the women the participant called as mother when they were growing up, if they had one; **Father means the men the participant called as father when they were growing up, if they had one.

### Childhood experience

From participants, 485 (78.9%) of respondents claim to have grown up with both parents as guardians. More than half, 343 (55.8%), of the study participants grew up in a house where the mother’s decision-making power is poor/low or in a house where the father is the head of the household. In this study, 39.5% of participants said large household purchase is done by father-only students whereas 76% of participants said it was their mothers who made small purchases most of the time. From study participants, 286 (46.5%) respondents reported that they had experienced at least one form of physical violence during their childhood. From these, 57 (9.3%) reported being threatened by a gun, knife, or wood at least once during their childhood.

### Behavioral related characteristics

Almost two-fifths of the participants 237 (38.7%) claimed that they had used alcohol (alcoholic beverage drinks including local alcohol) in their lifetime, out of these more than one-third 217 (35.3%) are current alcohol users, of which 17 (2.8%) use alcohol daily (Table 2).

**Table 2:** Behavioral characteristics of study participants at Jinka and Arba Minch University student Ethiopia, 2022.

Variables	Categories	Frequency (n)	Percent (%)
Ever used alcohol	Yes	237	38.7
	No	377	71.3
Current alcohol use (in the past 12 months)	Yes	217	35.3
	No	21	3.4
Frequency of alcohol use	Daily	17	2.8
	Once/twice per week	46	7.5
	Once/twice per month	69	11.2
	Once/twice per year	85	13.6
Ever used Khat*	Yes	129	30.5
	No	294	69.5
Current khat chewing (in the past 12 months)	Yes	48	7.8
	No	10	1.6
Ever used cigarette	Yes	36	5.9
	No	579	94.1
Current cigarette smoking (in the past 12 months)	Yes	27	4.4
	No	9	5.9

*Khat (*Catha edulis*) is a plant native to East Africa and the Arabian Peninsula, whose leaves are chewed for their stimulant effects.

### Attitude towards gender equality

Nearly half, 291 (47.3%), of the participants scored below the mean score of 7 and had poor attitudes towards gender equality. Out study participants, 98 (15.9%) of students do not believe all women are equal to men.

### Media-related characteristics

More than half of the participants 481 (78.2%) had information about gender equity in media. Of those, 149 (24.2%) of participants claim the school is the main source of information on gender equity. Furthermore, 220 (35.8%) were participating in youth clubs.

### Proportion of inequitable gender norms

In the current study, the mean score for the gender inequitable men scale was 61.4 (SD = ±8.5). From the study respondents, 271 participants, accounting for 44.1% (95% CI: 40.1, 48.1%), scored below 40 on the Gender Equitable Men scale, indicating adherence to gender inequitable norms.

### Item score of GEM scale for female and male students

In the Chi-square test, a statistically significance difference between male and female students was observed in the four domains. In the violence domain, almost half of the students, 48.7% female and 45.1% male students agreed either totally or partially that “a woman should tolerate violence to keep her family together.” Similarly, in the domestic and childcare domain more than half of students, 56.9% female and 61.4% male students, agreed either totally or partially that “a woman should obey her husband in all things.” In the reproductive and disease prevention domain, nearly one-third, 28.8% of female and 36.4% of male students agreed totally or partially that “it is a woman’s responsibility to avoid getting pregnant.” In the sexuality domain for the statement “men need more sex than women do” 43.9% of female and 49.0% of male students agreed either totally or partially (Supplementary file 1).

### Factors associated with inequitable gender norms

In the bivariable logistic regression analysis, several factors including sex, relationship status, field of study, year of study, residence before university enrollment, type of school attended before university, mother’s educational status, father’s educational level, experience of physical violence during childhood, Khat use, and attitude toward gender equality were found to be associated with inequitable gender norms at a significance level of *p* < 0.25. These factors were subsequently included in the multivariable logistic regression analysis to control for confounding effects. After adjusting for potential confounders in the multivariable logistic regression analysis, three factors remained statistically associated with favorable attitudes toward gender inequitable norms at a significance level of *p* < 0.05. These factors are as follows: The odds of holding inequitable gender norms were 1.75 times higher among male students compared to female students [Adjusted Odds Ratio (AOR) = 1.75; 95% CI: 1.19–2.56]. Individuals who had experienced a romantic relationship break-up had nearly two-fold higher odds of having inequitable gender norms compared to those who had not experienced (AOR = 2.10; 95% CI: 1.14–3.99). Finally participants with poor attitudes toward gender equality were found to have 3.14 times higher odds of holding inequitable gender norms (AOR = 3.14; 95% CI: 2.15–4.58) (Table 3).

**Table 3:** Bivariable and multivariable logistic regression analysis for factors associated with inequitable gender norms among Jinka and Arba Minch University students, Southern Ethiopia, 2022.

Variables	Gender norm		COR (95% CI)	*P*-value	AOR (95% CI)	*P*-value
	Inequitable n (%)	Equitable n (%)				
Sex						
Male	184 (48.9)	192 (51.1)	1.67 (1.20–2.33)	0.020	1.74 (1.19–2.56)	0.004
Female	87 (36.4)	152 (63.6)	1		1	
Relationship status						
In relationship	43 (37.4)	72 (62.6)	1		1	
Broken relationship	53 (57.6)	39 (42.4)	2.27 (1.30–3.98)	0.004	2.09 (1.14–3.88)	0.018
Never in relationship	135 (41.2)	193 (58.8)	1.17 (0.75–1.82)	0.480	0.96 (0.58–1.59)	0.890
Field of study						
Social	113 (52.8)	101 (47.2)	1		1	
Natural	158 (39.4)	234 (60.6)	0.58 (0.42–0.81)	0.001	0.76 (0.49–1.17)	0.220
Year of study						
First	40 (48.2)	43 (51.8)	0.86 (0.47–1.59)	0.640	1.28 (0.61–2.65)	0.510
Second	80 (40.4)	118 (59.6)	0.63 (0.37–1.06)	0.080	1.03 (0.52–1.93)	0.990
Third	109 (43.1)	144 (56.9)	0.70 (0.43–1.16)	0.170	0.89 (0.48–1.62)	0.690
Fourth and above	42 (51.9)	39 (48.1)	1		1	
Residence prior to University enrollment						
Urban	141 (38.2)	228 (61.8)	1		1	
Rural	130 (52.8)	116 (47.2)	1.81 (1.31–2.51)	<0.001	1.43 (0.89–2.27)	0.130
Types of secondary school attended						
Private	39 (36.4)	68 (63.6)	1		1	
Public	232 (45.7)	276 (54.3)	1.47 (0.95–2.25)	0.080	0.97 (0.56–1.69)	0.970
Mother educational status						
No formal education	97 (49.5)	99 (50.5)	1.71 (1.06–2.76)	0.030	0.98 (0.45–2.14)	0.950
Primary	102 (44.9)	125 (55.1)	1.43 (0.89–2.28)	0.140	1.11 (0.56–2.18)	0.760
Secondary	32 (39.0)	50 (61.0)	1.12 (0.62–2.02)	0.710	1.35 (0.66–2.75)	0.410
Above secondary	40 (36.4)	70 (63.6)	1		1	
Father educational status						
No formal education	76 (46.9)	86 (53.1)	1.77 (1.15–2.72)	0.010	1.42 (0.68–2.98)	0.350
Primary	86 (54.1)	73 (45.9)	2.36 (1.53–3.64)	<0.001	1.64 (0.85–3.16)	0.140
Secondary	46 (43.8)	59 (56.2)	1.56 (0.93–2.54)	0.080	1.12 (0.59–2.12)	0.730
Above secondary	63 (33.3)	126 (66.7)	1		1	
Physical violence experience during childhood						
Yes	141 (49.3)	145 (50.7)	1.48 (1.08–2.05)	0.020	1.32 (0.89–1.95)	0.160
No	130 (39.5)	199 (60.5)	1		1	
Attitude toward gender equality						
Poor	175 (60.1)	116 (39.9)	3.58 (2.56–2.05)	<0.001	3.14 (2.15–4.58)	<0.001
Good	96 (29.6)	228 (70.4)	1		1	
Ever used Khat						
Yes	34 (58.6)	24 (41.4)	1.91 (1.10–3.31)	0.020	1.57 (0.82–3.01)	0.170
No	237 (42.5)	320 (57.5)	1		1	

## Discussion

The study in Southern Ethiopia found that 44.1% of university students held inequitable gender norms. Being male, experiencing a romantic relationship breakup, and having poor attitudes toward gender equality were associated with these norms. Significant differences between male and female students were noted in endorsing inequitable norms in domains such as domestic life, childcare, reproductive health, and sexuality, except for violence domain.

In this study, the mean score for the Gender Equitable Men (GEM) scale was 61.38 (SD ±8.36), indicating that more than half of the study participants endorsed equitable gender norms. The higher scores observed could be attributed to the educational effect, as education has been shown to significantly influence gender socialization and attitudes towards gender norms (25–27, 38).

The study revealed that 44.1% of university students held inequitable gender norms, aligning with findings from a Congolese study 50% (38). This poses a public health challenge, as such norms influence health behaviors, reproductive health access, and gender-based violence.

In this study, differences were observed between male and female students in the domains of household and childcare, reproductive health and disease prevention, and sexuality regarding gender inequitable norms. This finding aligns with similar observations among university students in Turkey (28). In this study, male students endorsed inequitable gender norms in 23 out of 24 items. Reinforcing behaviors that drive gender-based violence, limiting access to essential health services, and perpetuate health disparities. In contrast, a study in Tanzania found that women endorsed gender inequitable norms in 21 out of 24 items (29). Additionally, a study from Congo showed that women endorsed more inequitable gender norms compared to men. They justified that women’s support for inequitable gender norms is influenced by their persistent experiences of unequal power dynamics (38). Partly, the differences between the current study and the studies in Tanzania could be attributed to methodological variations. The Tanzanian study focused on a project aimed at promoting men’s positive involvement in response to HIV/AIDS (29). Another possible explanation could be the difference in study populations. The latter study focused on the adult married population, where marriage can influence gender socialization towards more egalitarian views, especially among men. Recent research indicates that many husbands believe in equitable norms due to women’s increased workforce participation and the shared responsibility for household expenses (30, 31).

This study attested that a significant proportion of study participants accepted violence in intimate relationships, though there was no statistically significant difference based on sex. This acceptance poses as it normalizes gender-based violence, increases the risk of physical and mental health issues. This finding is in line with a study reported from Zambia (17). However, a study from Nigeria reported a statistically significant difference in participants’ acceptance of violence in intimate relationships based on sex (32). In this study, 37% of male and 36.4% of female students agreed either totally or partially with the statement “There are times when a woman deserves to be beaten.” A comparable proportion is reported from Uganda with 37% of females and 40% of males agreeing either totally or partially (33). Also, a study from Tanzania reported comparable proportions (34). However, a higher proportion was seen in Ethiopia, Zambia, and Congo (17, 23, 38). In the current study, 48.7% of male and 45.1% of female participants agreed either partial or total “a woman should tolerate violence to keep her family together.” Similarly, a study in Ethiopia reported 50% acceptance of violence in intimate relationships (23). In Congo, 49.2% of men and 29.3% of women participants were in agreement with the above statement (38).

University students’ acceptance of violence and inequitable gender norms suggests a tendency to internalize rather than challenge patriarchal views (35). This finding suggests that students’ acceptance of gender norms could lead to future engagement in or experience of Gender-Based Violence (GBV). Gender socialization, as theorized, begins early in life, shaping masculine and feminine identities through environmental influences, which solidify further during adolescence (9, 25, 36).

In our study, a notable gender difference was found in the acceptance of inequitable gender norms related to domestic and childcare responsibilities, with this domain showing the highest level of inequity compared to others. The unequal distribution of domestic duties can result in increased stress, mental health issues, and restricted access to resources, particularly for women, who often bear a disproportionate burden. This finding aligns with research from Zambia, which similarly reported high endorsement of traditional household role norms (17). The most supported inequitable belief in the domain of domestic life and childcare is that “women should obey their husbands in all matters.” Another study in Ethiopia found that 50% of respondents agreed with this statement (23). The finding is also consistent with a study reported from Zambia and Congo (17, 38). In this study, 52.7% of male and 41% of female students endorsed the belief that bathing and feeding children are solely the mother’s responsibility. However, higher endorsement rates were found in Zambia and Uganda (17, 33).

Support for inequitable gender norms attitude is also seen in the sexuality domain. In the two items, a significant difference is observed based on sex. However, studies from Nigeria and Uganda reported no difference (32). The most supported belief in the sexuality domain is that “men need more sex than women do,” endorsed by 49.0% of male and 43.9% of female students. From a public health standpoint, subservient norms restrict women’s agency in safe sexual practices and access to health services, which contributes to gender disparities in sexual health outcomes and mental health issues, while gendered beliefs encourage men to engage in risky sexual behaviors, raising the risk of STIs and unplanned pregnancies. Meanwhile, women, burdened by conforming to subservient norms, may face restricted choices and decision-making in their sexual lives (25).

In this study, a notable difference was found between sexes in the reproductive health and disease prevention domain. Specifically, 36.4% of male students and 28.8% of female students endorsed the statement “It is a woman’s responsibility to avoid getting pregnant.” This reflects ingrained gender norms that inhibit fair access to family planning resources, limit shared responsibility, and impose the burden of contraception on women. This finding aligns with a study conducted in Congo (38). In India, a similar report stated women are responsible for using contraceptives (34).

In the current study, male students had nearly twice the odds of endorsing inequitable gender norms compared to their female counterparts. This suggests that stereotypical attitudes towards gender equity remain prevalent among male students. This difference may be attributed to variations in the gender socialization process, where males are often taught and pressured from a young age to be strong, assertive, and dominant. Additionally, males who embrace and demonstrate equitable gender norms may face stigma and ridicule from their parents, peers, and society more so than females (25, 36, 37). This finding is confirmed by studies reported from Uganda, Turkey, India, Jordan, China, Mexico, and Europe (25, 28, 40–42). However, a study from Tanzania and Congo reported the odds of inequitable norms to be higher among women participants (34, 38). Another study from Tanzania negatively moderately correlated suggesting being a female leads to a decrease in the level of equity (29). Endorsing stereotypical gender norms that promote male dominance correlates with inequitable attitudes towards gender, including substance use, violence, delinquency, reduced male involvement in caregiving and household tasks, unsafe sexual practices, and perpetration of intimate partner violence (42).

In this study, students who have experienced broken romantic relationships show higher odds of endorsing inequitable gender norms. This could suggest that relationship dissolution may be linked to experiences of intimate partner violence (physical or sexual), influencing the process of gender socialization (32). Studies suggest relationship breakup results in violent behavior, decreased self-worth, and social respect (25, 43). Likewise, a study from Congo found that unmarried individuals, both women and men, scored high on the GEM scale, indicating they may not have experienced long-term serious relationships and thus have not been exposed to unequal power dynamics (38). Another study from Spain, reported males in intimate relationship experience predicted stronger endorsement of inequitable attitudes (44).

Participants with lower attitudes towards gender equality show a statistically significant association with favorable inequitable gender norms across natural equality, political participation, financial management, household activities, and education. Those with lower gender equality attitudes had nearly four times higher odds of endorsing favorable inequitable gender norms. Attitudes towards gender equality strongly influence gender norms socialization, potentially shaping acceptance of equitable gender norms based on negative attitudes towards gender equality. Findings from a study in Congo support these results, highlighting a robust link between attitudes towards gender equality and scores on the GEM scale (38). University students’ unequal gender norms could be addressed through curriculum-based programs that support gender equality, awareness campaigns that dispel preconceptions, and peer-led projects that foster discussion. Counseling for students with relationship problems, programs specifically designed for male students, and policy campaigning for gender-equal university laws are also crucial. To evaluate the effectiveness of these interventions and make appropriate strategy adjustments over time, ongoing research and monitoring are required.

### Strengths and limitations

This study among Ethiopian university students is one of the few assessing attitudes towards inequitable gender norms, with adequate representation of female students. The 95.0% response rate is another strength, indicating significant involvement and strengthening the validity of the results. The study’s generalizability is limited by its focus on only two universities which may not fully capture the cultural, social, and religious diversity. Caution is needed when interpreting the results, as the use of *p*-values to select variables in the statistical analysis can be sensitive to small sample variations. The study acknowledges a potential limitation due to the similarity of items in the “Inequitable gender norms” and “Gender equality attitudes” questionnaires, which may lead to some conceptual overlap. Other limitations include social desirability bias in sensitive questions about sexuality and substance use, and potential recall bias in remembering childhood experiences like physical violence and women’s autonomy. The study was conducted outside of class hours and was voluntary, with no incentives offered. Participation was completely voluntary because there were no consequences for not participating. The study also did not explore the impact of lecturers’ stereotypical or prejudiced attitudes on students.

## Conclusion

Establishing fair social norms is crucial for gender equality. This survey of University students found significant inequitable gender norm attitudes, particularly among men, on issues like violence, sexuality, home roles, and reproduction. From a public health perspective, these attitudes can perpetuate gender-based disparities, such as poor reproductive health, unequal healthcare access, and increased violence. Factors like sex, relationship experiences, and views on gender equality influence these norms. Public health interventions in universities are essential to challenge these norms, promote gender equity, and address long-term health and social impacts.

## Data Availability

The raw data supporting the conclusions of this article will be made available by the authors, without undue reservation.
