# Supplementary material for: Inequitable gender norms and its associated factors among university students in southern Ethiopia: a cross-sectional study, 2022
Source: Front Public Health. 2024 Dec 19;12:1462782. doi: 10.3389/fpubh.2024.1462782 (PMC11693685; doi:10.3389/fpubh.2024.1462782)
Supplement: Supplementary file 1 [file Table_1.DOCX]

**Supplementary file:** Scores of the Gender-Equitable Men Scale questions by sex of study participants at Jinka and Arba Minch University, Southern Ethiopia, 2022

| **Survey questions** | **Responses** | **Female** | | **Male** | | **P-value** |
| --- | --- | --- | --- | --- | --- | --- |
|  |  | **Total** | **Percent** | **Total** | **Percent** |  |
| **Violence Domain** | | | | | | |
| There are times when a woman deserves to be beaten | Totally agree | 31 | 13.0 | 57 | 15.2 | 0.72 |
|  | Partially agree | 56 | 23.4 | 82 | 21.8 |  |
|  | Disagree | 152 | 63.6 | 237 | 63.0 |  |
| A woman should tolerate violence in order to keep her family together | Totally agree | 57 | 23.8 | 112 | 29.8 | 0.27 |
|  | Partially agree | 51 | 21.3 | 79 | 18.9 |  |
|  | Disagree | 131 | 54.8 | 193 | 51.3 |  |
| It is okay for a man to hit his wife if she won’t have sex with him | Totally agree | 18 | 7.5 | 36 | 9.6 | 0.68 |
|  | Partially agree | 28 | 11.7 | 43 | 11.4 |  |
|  | Disagree | 193 | 80.8 | 297 | 79.0 |  |
| It is alright for a man to beat his wife if she is unfaithful | Totally agree | 31 | 13.0 | 62 | 16.5 | 0.27 |
|  | Partially agree | 40 | 16.7 | 73 | 19.4 |  |
|  | Disagree | 168 | 70.3 | 241 | 64.1 |  |
| If someone insults a man, he should defend his reputation with force | Totally agree | 21 | 8.8 | 40 | 10.6 | 0.72 |
|  | Partially agree | 31 | 13.0 | 51 | 13.6 |  |
|  | Disagree | 187 | 78.2 | 285 | 75.8 |  |
| If he has to a man using violence against his wife is a private matter that shouldn’t be discussed outside | Totally agree | 44 | 18.4 | 75 | 19.9 | 0.74 |
|  | Partially agree | 54 | 22.6 | 76 | 20.2 |  |
|  | Disagree | 141 | 59.0 | 225 | 59.0 |  |
| **Domestic Life and Childcare Domain** | | | | | | |
| A man should have the final word on decisions in his home | Totally agree | 45 | 18.8 | 102 | 27.1 | 0.05 |
|  | Partially agree | 68 | 28.5 | 91 | 24.2 |  |
|  | Disagree | 126 | 52.7 | 183 | 48.7 |  |
| A woman’s most important role is to take care of her home and cook for her family | Totally agree | 27 | 11.3 | 69 | 18.4 | 0.05 |
|  | Partially agree | 66 | 27.6 | 104 | 27.7 |  |
|  | Disagree | 146 | 61.1 | 203 | 54.0 |  |
| Giving the kids a bath and feeding the kids are only the mother’s responsibility | Totally agree | 38 | 15.9 | 62 | 16.5 | 0.02 |
|  | Partially agree | 62 | 25.9 | 136 | 36.2 |  |
|  | Disagree | 139 | 58.2 | 178 | 47.3 |  |
| A woman should obey her husband in all things | Totally agree | 67 | 28.0 | 114 | 30.3 | 0.54 |
|  | Partially agree | 69 | 28.9 | 117 | 31.1 |  |
|  | Disagree | 103 | 43.1 | 145 | 38.8 |  |
| A man should not take his child to the clinic without the child’s mother | Totally agree | 22 | 9.2 | 34 | 9.0 | 0.56 |
|  | Partially agree | 18 | 7.5 | 38 | 10.1 |  |
|  | Disagree | 199 | 83.3 | 304 | 80.9 |  |
| **Reproductive health and disease prevention Domain** | | | | | | |
| It is a woman’s responsibility to avoid getting pregnant. | Totally agree | 26 | 10.9 | 50 | 13.3 | 0.03 |
|  | Partially agree | 37 | 17.9 | 87 | 23.1 |  |
|  | Disagree | 176 | 73.6 | 239 | 63.6 |  |
| A real man produces a male child. | Totally agree | 17 | 7.1 | 33 | 8.8 | 0.21 |
|  | Partially agree | 14 | 5.9 | 35 | 9.3 |  |
|  | Disagree | 208 | 87.0 | 308 | 81.9 |  |
| Only when a woman has a child is she a real woman. | Totally agree | 13 | 7.1 | 30 | 8.0 | 0.02 |
|  | Partially agree | 12 | 5.9 | 41 | 10.9 |  |
|  | Disagree | 214 | 87.0 | 305 | 81.1 |  |
| Women who carry condoms on them are easy. | Totally agree | 25 | 10.5 | 41 | 10.9 | 0.42 |
|  | Partially agree | 31 | 13.0 | 63 | 16.8 |  |
|  | Disagree | 183 | 76.6 | 272 | 72.3 |  |
| A man should be outraged if his wife asks him to use a condom. | Totally agree | 16 | 6.7 | 22 | 5.9 | 0.66 |
|  | Partially agree | 24 | 10.0 | 46 | 12.2 |  |
|  | Disagree | 299 | 83.3 | 308 | 81.9 |  |
| Real men do not immediately go a doctor when they are sick | Totally agree | 14 | 5.9 | 34 | 9.0 | 0.048 |
|  | Partially agree | 17 | 7.1 | 44 | 11.7 |  |
|  | Disagree | 208 | 87.0 | 298 | 79.3 |  |
| **Sexuality Domain** | | | | | | |
| Men need more sex than women do | Totally agree | 50 | 20.9 | 83 | 22.1 | 0.44 |
|  | Partially agree | 55 | 23.0 | 101 | 26.9 |  |
|  | Disagree | 134 | 56.1 | 192 | 51.0 |  |
| You don’t talk about sex; you just do it | Totally agree | 31 | 13.0 | 36 | 9.6 | 0.29 |
|  | Partially agree | 35 | 14.6 | 67 | 17.8 |  |
|  | Disagree | 173 | 72.4 | 273 | 72.6 |  |
| Men are always ready to have sex | Totally agree | 35 | 14.6 | 65 | 17.3 | 0.32 |
|  | Partially agree | 68 | 28.5 | 88 | 23.4 |  |
|  | Disagree | 136 | 56.5 | 223 | 59.3 |  |
| It is the man who decides when to have sex with a partner | Totally agree | 17 | 7.1 | 26 | 6.9 | 0.002 |
|  | Partially agree | 20 | 8.4 | 70 | 18.6 |  |
|  | Disagree | 202 | 84.5 | 280 | 74.5 |  |
| Men need other women even if the things with his wife are fine | Totally agree | 14 | 5.9 | 27 | 7.2 | 0.24 |
|  | Partially agree | 19 | 7.9 | 44 | 11.7 |  |
|  | Disagree | 206 | 86.2 | 305 | 81.1 |  |
| Employed women do not make a good wife | Totally agree | 13 | 5.4 | 23 | 6.1 | 0.64 |
|  | Partially agree | 21 | 8.8 | 41 | 10.9 |  |
|  | Disagree | 205 | 85.8 | 312 | 83.0 |  |
| A woman should not initiate sex | Totally agree | 16 | 6.7 | 51 | 13.6 | 0.03 |
|  | Partially agree | 31 | 13.0 | 49 | 13 |  |
|  | Disagree | 192 | 80.3 | 276 | 73.4 |  |
